# Supplementary material for: Comprehensive Comparison of the Capacity of Functionalized Sepharose, Magnetic Core, and Polystyrene Nanoparticles to Immuno-Precipitate Procalcitonin from Human Material for the Subsequent Quantification by LC-MS/MS
Source: Int J Mol Sci. 2023 Jun 30;24(13):10963. doi: 10.3390/ijms241310963 (PMC10341910; doi:10.3390/ijms241310963)
Supplement: Supplementary file 1 [file ijms-24-10963-s001.zip › ijms-2459025-supplementary.docx]

Supplementary data, Masetto, et al.

Comprehensive comparison of the capacity of functionalized Sepharose, magnetic core, and polystyrene nanoparticles to immuno-precipitate procalcitonin from human material for the subsequent quantification by LC-MS/MS

**Figure S1.** Experimental setting of used particles. Typical experiment reflecting used particle amounts of PGS, MagP, and Lx particles in a 1.5 ml Eppendorf tube. Due to transparency and low amount of Sepharose particles (1), no clear pellet is visible. On the other hand, MagP (2) and Lx (3) show a compact pellet on the bottom of the working tubes.

**Figure S2.** Amino acid sequences of highly purified recombinant human PCT (rhPCT) were used in this work. The amino acid sequence is derived from the manufacturer HyTest Ltd (Turku, Finland), according to the MALDI-MS analysis of the technical note. The rhPCT contains full-length PCT with an additional methionine residue at the N-terminus (Ala1-Asn116, Sequence 1) and partially truncated PCT, lacking the first alanine (Pro2-Asn116, Sequence 2). The reference sequence of human PCT corresponds to the UniProt P01258 entry, without the signal peptide. The identical amino acids of the three sequences are highlighted in grey, while those similar in two of the three sequences are shown in light blue. The yellow box indicates the portion of the Calcitonin moiety possibly involved in forming a disulfide bridge. The green bars show the tryptic peptides used for quantification in the mass spectrometry method of this work. Putative oxidized methionine residues are indicated by an asterisk.

**Table S1.** iBright quantification of the rhPCT precipitated by the three particle kinds. Data referring to Figure 3, lanes 4 – 9, in the Manuscript. The depletion efficiency (%) is reported in parenthesis after the net concentration recovery (in ng).

|  | Control | **PGS**  **(Lane 4 / 5)** | | **MagP**  **(Lane 6 / 7)** | | **Lx**  **(Lane 8 / 9)** | |
| --- | --- | --- | --- | --- | --- | --- | --- |
| Value measured at iBright | 1.000 | 0.568 | 0.498 | 1.111 | 1.100 | 0.771 | 0.822 |
| PCT Ag [ng] / band | 500 | 284.0 | 249.0 | 555.5 | 550.0 | 385.5 | 411.0 |
| Mean of PCT Ag [ng] | 500 | 266.5 (53.3%) | | 552.8 (110.6%) | | 398.3 (79.7%) | |

**Table S2.** Quantitation of the rhPCT, remaining in the supernatant after 2 h, 4 h, or 6 h depletion with the respective immuno-functionalized nano-particles (PGS, MagP, Lx). The recovery in percentage (%), expressing the trueness of pull-down efficiency, is reported in parenthesis after the net concentration recovery (in ng/mL). The concentrations of spiked rhPCT (0.5 ng/ml, 5 ng/ml, and 50 ng/ml) were chosen as they cover the whole analytical range of the commonly used PCT immunoassays. The elevated amount of rhPCT, recovered after 2 h incubation with PGS, is due to difficulties in the handling of this material, which can partially remain in suspension and may interfere with subsequent analysis. Quantitation was carried out with the immunoturbidimetric assay PCT FS (DiaSys Diagnostic Systems, Holzheim, Germany).

|  |  | **Incubation time [h]** | | | |
| --- | --- | --- | --- | --- | --- |
| Particle type | Solution PCT | 0 | 2 | 4 | 6 |
| **PGS** | 0.5 ng/ml | 0.5 | 1.361 (-172.2%) | 0.117 (76.6%) | 0.349 (30.2%) |
|  | 5 ng/ml | 5 | 24.41 (-388.2%) | 6.64 (-32.8%) | 5.57 (-11.4%) |
|  | 50 ng/ml | 50 | 83.86 (-67.7%) | 17.45 (65.1%) | 25.81 (48.4%) |
| **MagP** | 0.5 ng/ml | 0.5 | 0.01 (98.0%) | 0.00 (100%) | 0.00 (100%) |
|  | 5 ng/ml | 5 | 0.04 (99.2%) | 0.00 (100%) | 0.01 (99.8%) |
|  | 50 ng/ml | 50 | 0.12 (99.8%) | 0.03 (99.9%) | 0.01 (100%) |
| **Lx** | 0.5 ng/ml | 0.5 | 0.06 (88.0%) | 0.02 (96.0%) | 0.02 (96.0%) |
|  | 5 ng/ml | 5 | 0.04 (99.2%) | 0.02 (99.6%) | 0.02 (99.6%) |
|  | 50 ng/ml | 50 | 0.11 (99.8%) | 0.06 (99.9%) | 0.04 (99.9%) |

**Figure S3 (a-h).** PCT is sensitive to oxidation. The same quantity of rhPCT was incubated with increasing amounts of DTT (1 to 5 mmol) and incubated at 37 °C for 6 or 10 days. The recovery (%) of rhPCT was measured with a mAb-based immuno-assay (BRAHMS-Roche CLIA, Figures **a-d**) and a pAb-based immuno-assay (DiaSys PETIA, Figures **e-h**) and set about an untreated sample. The loss in immuno-reactivity can be partially avoided upon the addition of DTT. The pAb-based immuno-assay reveals a lower sensitivity to oxidation compared to the mAb-based one.

**Figure S4 (a,b).** Assessment of the saturation of Fc-fragments on immuno-functionalized nano-particles. (**a**) PGS nano-particles were immuno-functionalized by covalent cross-link with PCT-specific polyclonal antibodies (lanes 4 and 5) and subsequently incubated with human IgG Fc-fragments (50 µg), to assess saturation of free Protein G binding sites. Blocking of immuno-functionalized nano-particles by human Fc-fragments significantly reduces the binding of immunoglobulins to protein G or an unspecific interaction of rheumatoid factors (unspecific Fc-Fc interactions by unrelated human immunoglobulins), derived from human serum samples. Lanes 6 and 7 show crosslinking of antibodies without further incubation with Fc-fragments. Lane 2 shows the input of pAb and lane 3 input of Fc-fragments. (**b**) Immuno-functionalized MagP were used to assess saturation of Fc-fragments on this type of particle. Lane 4 shows the MagP incubated with Fc-fragments alone. Lanes 5 to 9 show MagP incubated with increasing amounts of anti-PCT pAbs and 50 µg Fc-fragments. Lane 2 shows the input of pAb and lane 3 input of Fc-fragments. As evident by SDS-PAGE and Coomassie staining in this setting, significantly fewer Fc-fragments bind to the functionalized MagP nano-particles. This is probably due to the much lower content of protein G of MagP, in comparison to PGS.
